# Identification of Factors Contributing to Methadone-Induced Daytime Sleepiness in Cancer Patients and Proposal of the Conversion Ratio from Other Opioids to Oral Methadone: A Retrospective Cohort Study

**DOI:** 10.1089/pmr.2023.0007

**Published:** 2023-07-28

**Authors:** Miho Takemura, Kazuyuki Niki, Yoshiaki Okamoto, Yoshinobu Matsuda, Makie Kohno, Mikiko Ueda

**Affiliations:** ^1^ Department of Clinical Pharmacy Research and Education, Osaka University Graduate School of Pharmaceutical Sciences, Suita, Japan.; ^2^ Department of Pharmacy, Ashiya Municipal Hospital, Ashiya, Japan.; ^3^ Department of Palliative Care, Ashiya Municipal Hospital, Ashiya, Japan.

**Keywords:** cancer pain management, drug-induced daytime sleepiness, methadone, opioid switch, palliative care

## Abstract

**Background:**

When methadone is used to treat cancer pain, the Japanese health insurance system recommends to determine the starting dose according to the equivalency conversion table based on the morphine-equivalent daily dose (MEDD) of prior opioids proposed by the National Comprehensive Cancer Network. Owing to the wide range in variability of the conversion table, methadone increases the incidence of daytime sleepiness.

**Objective:**

To identify the factors associated with daytime sleepiness and propose a conversion ratio from pretreatment MEDD to oral methadone that decreases the risk of daytime sleepiness.

**Design:**

Retrospective cohort study.

**Setting/Subjects:**

One hundred patients who started oral methadone to relieve cancer pain at Ashiya Municipal Hospital (Hyogo, Japan) from January 1, 2013, to August 31, 2022, were enrolled.

**Measurements:**

The primary endpoint, the conversion ratio from pretreatment MEDD to oral methadone without daytime sleepiness, was determined using receiver operator characteristic (ROC) curve analysis.

**Results:**

The incidence of daytime sleepiness within seven days of methadone initiation was 40.0%. The factors identified as contributing to daytime sleepiness were pretreatment MEDD (odds ratio [OR]: 0.941, 95% confidence interval [CI]: 0.916–0.966, *p* <0.001) and methadone dose (OR: 1.395, 95% CI: 1.178–1.652, *p* <0.001). The conversion ratio from pretreatment MEDD to oral methadone was 0.24, with an area under the ROC curve of 0.909 (*p* <0.001).

**Conclusions:**

Daytime sleepiness developed when methadone dose is high relative to pretreatment MEDD. To the best of our knowledge, this is the first study to suggest the conversion ratio from pretreatment MEDD to oral methadone without causing daytime sleepiness.

## Background

Opioids have μ-opioid receptor-stimulating effects, which play an important role in the development of analgesia, daytime sleepiness, and tolerance to opioids.^1–3^ Daytime sleepiness is a clinical problem because it is associated with significantly decreased physical activity and cognitive function, thereby disrupting daily activities. Methadone antagonizes the N-methyl-D-aspartate (NMDA) receptor, resulting in an analgesic effect on many clinical conditions, including neuropathic pain and hyperalgesic states.^4^ However, NMDA receptor antagonists have been shown to inhibit opioid tolerance. Because methadone has NMDA antagonist activity, it could be effective in restricting the development of tolerance.^5^ Notably, a Cochrane review reported that methadone can induce a higher incidence of daytime sleepiness than morphine.^6^

Although previous studies did not find a correlation between the incidence of daytime sleepiness and methadone dose,^7–9^ all these studies involved patients on methadone maintenance treatment; thus, these patients had been receiving methadone for at least some months and had already reached a stable maintenance methadone dose at the start of observation. However, sleepiness that develops within a few days after methadone initiation may involve central sleep apnea, characterized by decreased responsiveness to hypercapnia in the chemoreceptors.^10–12^ As the risk of central sleep apnea occurs in a dose-dependent manner, it is possible that daytime sleepiness may similarly develop in response to methadone dose.^10,11^

When methadone is used to treat cancer pain, the Japanese health insurance system recommends to determine the starting dose according to the equivalency conversion table based on the morphine-equivalent daily dose (MEDD) of prior opioids proposed by the National Comprehensive Cancer Network (NCCN). Owing to the wide range in the equivalency conversion table, methadone may be overdosed during switching, leading to an increased incidence of daytime sleepiness. However, to our knowledge, no previous study has investigated the factors affecting the onset of daytime sleepiness in patients initiated on methadone for cancer pain relief. We have initiated methadone for cancer pain relief in many patients and thus have observed a large number of cases in which daytime sleepiness developed after methadone initiation.

## Objective

We aimed to identify the factors associated with daytime sleepiness and propose a conversion ratio from pretreatment MEDD to oral methadone that decreases the risk of daytime sleepiness.

## Design

The following data were collected from medical records: age, sex, body mass index, Eastern Cooperative Oncology Group performance status (ECOG PS), primary cancer site, type of pain, pretreatment opioids and their MEDD, methadone dose, patterns of methadone introduction (stop-and-go strategy, three-days switch strategy, or add-on therapy), laboratory values, and concomitant medications at the start of methadone administration.

Daytime sleepiness was evaluated according to the patients' complaints and medical records by physicians, nurses, and pharmacists. Daytime sleepiness was defined as the onset or worsening of daytime sleepiness after methadone initiation. A previous study reported that the incidence of adverse events was highest within seven days after methadone initiation; therefore, the observation period in this study was seven days after methadone initiation.^13^

Pain intensity, as reported by the patients, was based on the numerical rating scale (NRS) or verbal rating scale (VRS) and obtained at baseline and after the administration of oral methadone (on days 1, 2, 3, 5, and 7). NRS and VRS scores were converted to number 0–3 as follows: 0 (NRS 0, no pain); 1 (NRS >0–4, mild pain); 2 (NRS >4–7, moderate pain); and 3 (NRS >7–10, severe pain).^14^ We also investigated usage count of opioid rescue medications at baseline and after the administration of oral methadone (on days 1, 2, 3, 5, and 7).

To clarify the factors affecting the onset of daytime sleepiness following methadone initiation, a multivariate logistic regression analysis was performed. We used daytime sleepiness (1 = with and 0 = without) as the dependent variable and considered the following potential confounding factors: age, sex, ECOG PS, pretreatment MEDD, methadone dose, patterns of methadone introduction, pain score at baseline, and usage count of opioid rescue medications at baseline. We also compared pretreatment MEDD, methadone dose, patterns of methadone introduction, changes in pain intensity and usage count of opioid rescue medications at baseline and after methadone administration, and baseline patient characteristics between patients with and without daytime sleepiness within seven days after methadone initiation to investigate the safe and efficacious methadone initiation methods.

The cutoff conversion ratio from pretreatment MEDD to oral methadone without daytime sleepiness was determined using receiver operator characteristic (ROC) curve analysis for patients switching from prior opioids to oral methadone. A multivariate logistic regression model was developed with daytime sleepiness as the dependent variable and the ratio of oral methadone dose to pretreatment MEDD as the independent variable. The conversion ratio from other opioids to oral methadone was defined as a 24-hour oral methadone dose/pretreatment MEDD for each patient.^15^

## Settings/Subjects

Patients undergoing palliative treatment who started oral administration of methadone for cancer pain at Ashiya Municipal Hospital (Hyogo, Japan) from January 1, 2013, to August 31, 2022, were enrolled. Patients who had received methadone therapy at other hospitals were excluded.

## Measurements

The primary endpoint was the conversion ratio from pretreatment MEDD to oral methadone without daytime sleepiness. The secondary endpoints were the incidence of daytime sleepiness within seven days after starting methadone, the factors influencing daytime sleepiness, and their odds ratios (ORs).

## Statistics

A multivariate logistic regression model was used to identify the factors affecting the onset of daytime sleepiness following methadone initiation. Pretreatment MEDD and methadone doses in patients with and without daytime sleepiness were compared using the Student's *t*-test. All statistical analyses were performed using BellCurve for Excel (Social Survey Research Information Co., Ltd., Tokyo, Japan), and *p*-values <0.05 were considered statistically significant.

## Ethical Approval

This study was conducted in accordance with the ethical principles for medical research outlined in the Declaration of Helsinki (1964) and approved by the Ethical Review Board of Ashiya Municipal Hospital (IRB approval code no. 76). Written consent for publication of this study was obtained from all patients.

## Results

One hundred and eight patients who started oral methadone treatment for cancer pain were identified during the study period. Eight patients who had received methadone treatment at other hospitals were excluded. Consequently, 100 patients were included in this study. Daytime sleepiness was reported in 40.0% of patients within seven days after the start of methadone treatment. The onset of daytime sleepiness was observed after an average of 2.05 days from the start of methadone administration. The baseline characteristics of patients with daytime sleepiness (*n* = 40) and those without daytime sleepiness (*n* = 60) are shown in Table 1. There was no significant difference in any of the characteristics between the two groups.

**Table 1.** Baseline Patient Characteristics

	*Patients with daytime sleepiness (*n* = 40)*	*Patients without daytime sleepiness (*n* = 60)*	*p*
Age (years), mean ± SD	69.4 ± 13.5	66.0 ± 13.8	0.174^a^
Sex, male, *n* (%)	18 (45.0)	23 (38.3)	0.323^b^
BMI (kg/m^2^), mean ± SD	19.7 ± 3.9	19.3 ± 3.4	0.627^a^
ECOG PS, *n* (%)			
4	10 (25.0)	11 (18.3)	0.478^b^
3	11 (27.5)	24 (40.0)	
2	13 (32.5)	14 (23.3)	
≤1	6 (15.0)	11 (18.3)	
Primary cancer site, *n* (%)			
Lung	9 (22.5)	6 (10.0)	0.408^b^
Colon	7 (17.5)	11 (18.3)	
Pancreas	5 (12.5)	10 (16.7)	
Uterine	4 (10.0)	6 (10.0)	
Prostate	3 (7.5)	1 (1.7)	
Breast	2 (5.0)	6 (10.0)	
Others	10 (25.0)	20 (33.3)	
Type of pain, *n* (%) (including duplicate answers)			
Somatic pain	28 (70.0)	42 (70.0)	0.590^b^
Visceral pain	13 (32.5)	25 (41.7)	0.238^b^
Neuropathic pain	30 (75.0)	42 (70.0)	0.378^b^
Pretreatment opioids, *n* (%)			
Hydromorphone	14 (35.0)	11 (18.3)	0.085^b^
Morphine	9 (22.5)	7 (11.7)	
Fentanyl	8 (20.0)	15 (25.0)	
Tapentadol	4 (10.0)	10 (16.7)	
Others	5 (12.5)	17 (28.3)	
Laboratory values at baseline, median (IQR)			
AST (U/L)	27.0 (11.0–162.0)	21.5 (10.0–133.0)	0.417^c^
ALT (U/L)	19.0 (4.0–142.0)	14.0 (5.0–153.0)	0.200^c^
γ-GTP (U/L)	65.0 (7.0–768.0)	49.0 (7.0–944.0)	0.558^c^
Scr (mg/dL)	0.67 (0.20–1.82)	0.66 (0.28–1.95)	0.796^c^
eGFR (mL/minute)	81.4 (21.5–324.4)	77.2 (16.3–254.1)	0.348^c^
BUN (mg/dL)	16.4 (5.4–50.0)	16.0 (5.9–50.1)	0.861^c^
K^+^ (mg/dL)	4.2 (3.4–6.4)	4.4 (1.9–6.6)	0.282^c^
Ca^2+^ (mg/dL)	8.8 (7.5–10.3)	8.4 (7.2–10.5)	0.350^c^
Mg^2+^ (mg/dL)	2.1 (1.6–2.5)	2.1 (1.6–2.5)	0.734^c^
Concomitant medications (including duplicate answers), *n* (%)			
Acetaminophen	10 (25.0)	12 (20.0)	0.800^b^
NSAIDs	17 (42.5)	28 (46.7)	0.419^b^
Adjuvant analgesics	19 (47.5)	39 (65.0)	0.063^b^
Corticosteroids	16 (40.0)	31 (51.7)	0.174^b^
Gabapentinoids	4 (10.0)	6 (10.0)	0.639^b^
SNRI	4 (10.0)	5 (8.3)	0.520^b^
Others	3 (7.5)	5 (8.3)	0.597^b^

^a^ Student's *t* test.

^b^ Chi-square for independence test.

^c^ Mann–Whitney's *U* test.

γ-GTP, γ-glutamyl transpeptidase; ALT, alanine transaminase; AST, aspartate transaminase; BMI, body mass index; BUN, blood urea nitrogen; ECOG PS, Eastern Cooperative Oncology Group Performance Status; eGFR, estimated glomerular filtration rate; IQR, interquartile range; NSAIDs, nonsteroidal anti-inflammatory drugs; Scr, serum creatinine; SD, standard deviation; SNRI, serotonin-norepinephrine reuptake inhibitor.

A multivariate logistic regression analysis showed that pretreatment MEDD (OR: 0.941, 95% confidence interval (CI): 0.916–0.966, *p* <0.001) and methadone dose (OR: 1.395, 95% CI: 1.178–1.652, *p* <0.001) were the significant factors that affected the incidence of daytime sleepiness within seven days after the start of methadone treatment (Table 2).

**Table 2.** A Multivariate Logistic Regression Analysis of Daytime Sleepiness Following Methadone Administration

Variables	OR	95% CI	*p*
Age (years)	0.998	0.942–1.056	0.933
Sex	0.698	0.189–2.571	0.588
ECOG PS	1.171	0.622–2.204	0.626
Pretreatment MEDD (mg/day)	0.941	0.916–0.966	<0.001
Methadone dose (mg/day)	1.395	1.178–1.652	<0.001
Patterns of methadone introduction	1.155	0.001–1.820	0.742
Pain score at baseline	0.727	0.301–1.757	0.479
Usage count of opioid rescue medications at baseline	1.334	0.900–1.976	0.151

CI, confidence interval; MEDD, morphine-equivalent daily dose; OR, odds ratio.

Table 3 shows the pretreatment MEDD and the methadone dose in patients with and without daytime sleepiness. The MEDD of the previously administered opioids was significantly lower in patients with daytime sleepiness (67.4 ± 62.8 mg/day) than in those without daytime sleepiness (195.1 ± 221.1 mg/day, *p* <0.001). Moreover, the rate of patients using pretreatment MEDD <60 mg/day was significantly higher in patients with daytime sleepiness (72.5%) than in those without daytime sleepiness (5.0%). There was no significant difference in the methadone dose between patients with daytime sleepiness (17.0 ± 8.8 mg/day) and without daytime sleepiness (19.3 ± 11.9 mg/day, *p* = 0.587). The proportion of patients whose methadone dose was higher than the dose converted from the pretreatment MEDD proposed by the NCCN was 17.5% in patients with daytime sleepiness compared to 0.0% in patients without daytime sleepiness.

**Table 3.** Comparison of Methadone Induction Methods in Patients With and Without Daytime Sleepiness

	Patients with daytime sleepiness (*n* = 40)	*Patients without daytime sleepiness (*n* = 60)*	*p*
Pretreatment MEDD (mg/day), mean ± SD	67.0 ± 62.8	194.0 ± 221.1	<0.001^a^
Hydromorphone	54.2 ± 42.3	93.5 ± 43.3	0.034^a^
Morphine	134.9 ± 90.3	409.1 ± 330.8	0.031^a^
Fentanyl	54.2 ± 29.6	160.9 ± 132.5	0.007^a^
Tapentadol	30.0 ± 15.0	119.0 ± 72.6	0.004^a^
Others	30.8 ± 20.3	243.6 ± 291.6	0.008^a^
Methadone dose (mg/day), mean ± SD	17.0 ± 8.8	19.3 ± 11.9	0.587^a^
Overdose rather than MEDD conversion strategy suggested by the NCCN, *n* (%)	7 (17.5)	0 (0.0)	0.001^b^
Add on methadone to pretreatment opioids, *n* (%)	4 (10.0)	5 (8.3)	0.520^b^
Add on 5 mg/day methadone	0 (0.0)	3 (5.0)	
Add on 10 mg/day methadone	0 (0.0)	2 (3.3)	
Add on 15 mg/day methadone	3 (7.5)	0 (0.0)	
Add on 30 mg/day methadone	1 (2.5)	0 (0.0)	
Discontinued methadone or reduced their methadone dose due to adverse events, *n* (%)	10 (25.0)	0 (0.0)	<0.001^b^
Excessive daytime sleepiness	6 (15.0)	0 (0.0)	
Oversedation	3 (7.5)	0 (0.0)	
Delirium	1 (2.5)	0 (0.0)	

^a^ Student's *t* test.

^b^ Chi-square for independence test.

NCCN, National Comprehensive Cancer Network.

Regarding the patterns of methadone introduction, four patients (10.0%) with daytime sleepiness and five patients (8.3%) without daytime sleepiness started methadone as an add-on to ongoing opioid treatment. The methadone dose added to pretreatment opioids was lower in patients without daytime sleepiness than in those with daytime sleepiness (three patients added 5 mg/day and two patients added 10 mg/day methadone), whereas patients with daytime sleepiness received higher doses of methadone (three patients added 15 mg/day and one patient added 30 mg/day methadone). Regarding adverse events, 10 patients (25.0%) with daytime sleepiness discontinued or decreased their methadone dose: six patients (15.0%) due to excessive daytime sleepiness, three patients (7.5%) due to oversedation, and one patient (2.5%) due to delirium. In contrast, none of patients (0.0%) who did not experience daytime sleepiness discontinued or reduced their methadone dose.

**FIG. 1.**
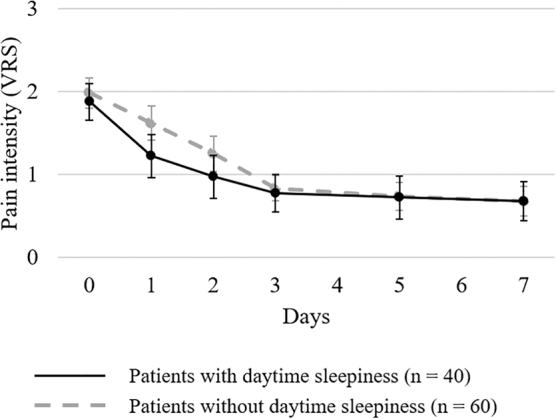
Change in the mean VRS scores on pain. The VRS scores were determined at baseline and on days 1, 2, 3, 5, and 7 after starting methadone administration in patients with and without daytime sleepiness. NRS and VRS scores were converted to numbers of 0–3 as follows: 0 (NRS 0, no pain); 1 (NRS >0–4, mild pain); 2 (NRS >4–7, moderate pain); and 3 (NRS >7–10, severe pain).^14^ NRS, numerical rating scale; VRS, verbal rating scale.

The changes in the mean pain intensity at baseline and after administration of methadone in patients with and without daytime sleepiness are shown in Figure 1. At baseline, the mean pain intensities (95% CI) in patients with and without daytime sleepiness were 1.88 (1.65–2.10) and 1.98 (1.80–2.17), respectively, with the difference not being statistically significant (*p* = 0.462). The reduction in pain intensity on day one was significantly higher in patients with daytime sleepiness than in those without daytime sleepiness (*p* = 0.013), although no statistically significant difference was observed in the reduction of pain intensity on days 2, 3, 5, and 7 between the two groups (day two, *p* = 0.137; day three, *p* = 0.864; day five, *p* = 0.780; and day seven, *p* = 0.994).

**FIG. 2.**
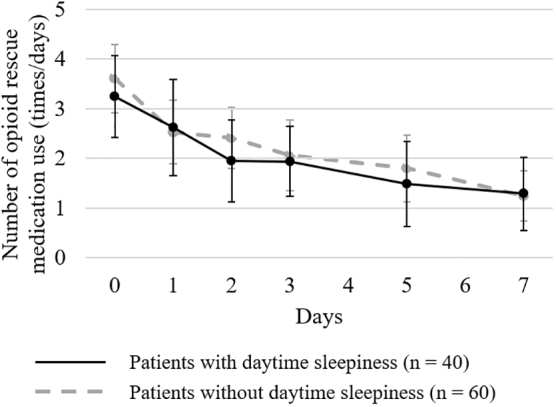
Change in the mean use of opioid rescue medications. The usage counts of rescue medications were investigated at baseline and on days 1, 2, 3, 5, and 7 after starting methadone administration in patients with and without daytime sleepiness. When multiple types of rescue medications were used, the total usage counts were determined.

The transition in the usage count of opioid rescue medications is shown in Figure 2. There was no significant difference in the average usage count of opioid rescue medications at the start of methadone administration (day zero) between patients with daytime sleepiness (mean, 3.24; 95% CI, 2.42–4.07) and those without daytime sleepiness (mean, 3.61; 95% CI, 2.92–4.30, *p* = 0.393). The average usage counts of opioid rescue medications on day seven significantly decreased compared to those at baseline in both patients with daytime sleepiness (*p* <0.001) and those without daytime sleepiness (*p* <0.001). A comparison of the decrease in the average use of opioid rescue medications between the two groups revealed no significant difference (day one, *p* = 0.776; day two, *p* = 0.493; day three, *p* = 0.642; day five, *p* = 0.588; and day seven, *p* = 0.522).

Table 4 shows the seven patients who switched from pretreatment MEDD of <60 mg/day to methadone. Four patients were switched to 5 mg/day of methadone, including one patient who had never used any opioid (her pretreatment MEDD was 0 mg/day). Three patients switched to 10 mg/day of methadone from prior opioids. Daytime sleepiness occurred in three patients who switched to 10 mg/day methadone and in one patient who had never used any opioid. None of the other three patients (who switched from prior opioids to 5 mg/day methadone) experienced daytime sleepiness. In all seven patients, pain intensities decreased to less than half of those at baseline within seven days after the start of methadone treatment.

**Table 4.** Seven Patients Switched from Pretreatment Morphine-Equivalent Daily Dose <60 mg/day to Low-Dose Methadone

Patient number	Pretreatment opioids	Methadone dose	Daytime sleepiness	>50% pain relief
1	Oral hydromorphone 6 mg/day (MEDD 30 mg/day)	5 mg/day	–	+
2	Transdermal fentanyl 1 mg/day (MEDD 30 mg/day)	5 mg/day	–	+
3	Intravenous morphine 24 mg/day (MEDD 48 mg/day)	5 mg/day	–	+
4	Oral hydromorphone 10 mg/day (MEDD 50 mg/day)	10 mg/day	+	+
5	Oral hydromorphone 6 mg/day (MEDD 30 mg/day)	10 mg/day	+	+
6	Oral tapentadol 100 mg/day (MEDD 30 mg/day)	10 mg/day	+	+
7	Opioid naive (MEDD 0 mg/day)	5 mg/day	+	+

**FIG. 3.**
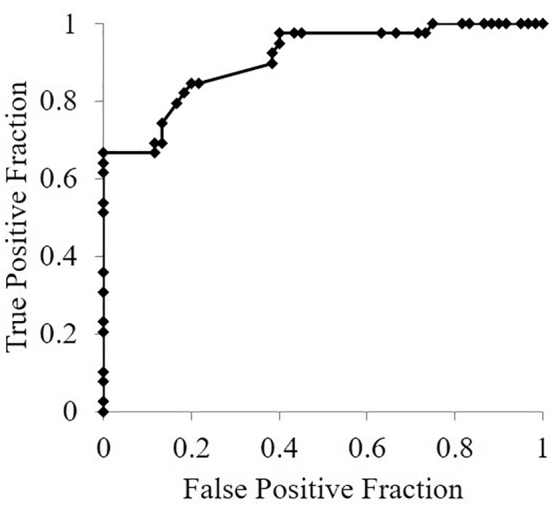
Receiver operating characteristic curve for detecting the threshold for conversion ratios of methadone daily dose/pretreatment MEDD without daytime sleepiness. Daytime sleepiness was defined as the dependent variable and the ratio of oral methadone daily dose/pre-treatment MEDD was defined as the independent variable. MEDD, morphine-equivalent daily dose.

ROC curve analysis (Fig. 3) was performed to obtain the cutoff value of the conversion ratio from pretreatment MEDD to oral methadone without daytime sleepiness. The best cutoff value of the 24-hour oral methadone dose/pretreatment MEDD was 0.24 (area under the ROC curve: 0.909, *p* <0.001), suggesting that 1 mg of pretreatment MEDD is equivalent to 0.24 mg of oral methadone.

## Discussion

To our knowledge, this is the first study to clarify the factors affecting the onset of daytime sleepiness in patients initiated on methadone for cancer pain relief and to suggest the conversion ratio from pretreatment MEDD to oral methadone without daytime sleepiness. The incidence of daytime sleepiness within seven days after methadone initiation was 40.0%; the onset of daytime sleepiness was observed after an average of 2.05 days from the start of methadone administration. A multivariate logistic regression analysis identified pretreatment MEDD and the methadone dose as contributing factors to daytime sleepiness. The MEDD of previously administered opioids was significantly lower in patients with daytime sleepiness than in those without daytime sleepiness; the methadone dose tended to be higher in patients with daytime sleepiness than in those without daytime sleepiness. These results suggest that daytime sleepiness may occur when the methadone dose is high relative to pretreatment MEDD.

The proportion of patients who were overdosed more than the dose suggested by the NCCN was 17.5% in patients with daytime sleepiness, compared to 0.0% in patients without daytime sleepiness. The methadone dose added to ongoing opioid treatment was higher in patients with daytime sleepiness than in those without daytime sleepiness, although the rate of patients who started methadone as an add-on to ongoing opioid treatment was similar between the two groups. Furthermore, in the seven patients who switched from pretreatment MEDD <60 mg/day to methadone, four patients with high methadone dose relative to their pretreatment MEDD developed daytime sleepiness within seven days, and the other three patients with low methadone dose relative to their pretreatment MEDD did not experience daytime sleepiness. These results also indicate that daytime sleepiness may develop at a higher methadone dose for pretreatment MEDD.

In patients with daytime sleepiness, 33 (82.5%) of the 40 patients developed daytime sleepiness, although their methadone doses were determined according to the conversion table based on the pretreatment MEDD proposed by the NCCN. This may be due to the wide range of the conversion table, which can result in an overdose of methadone. Therefore, we identified the cutoff value of the conversion ratio for switching from prior opioids to oral methadone without the onset of daytime sleepiness. Two patients on 30 mg/day pretreatment MEDD and one patient on 48 mg/day pretreatment MEDD were able to safely switch to 5 mg/day methadone without daytime sleepiness.

Although the administration of methadone to patients with a pretreatment MEDD of <60 mg/day is generally prohibited in Japan, the aforementioned three cases suggest that methadone may be safely introduced to patients with a pretreatment MEDD of <60 mg/day. If a patient switches from pretreatment MEDD <20 mg/day (e.g., 0.5 mg/day fentanyl tape or 4 mg/day hydromorphone tablet) to oral methadone, the methadone dose will be <5 mg/day according to the conversion ratio suggested in this study. Since the 1 or 10 mg/mL oral solution is unavailable in Japan, a low-dose methadone formulation, such as a 1 mg tablet, would be necessary.

In this study, for the patients who had used 12 and 14 mg/day hydromorphone tablets, methadone was started at 15 mg/day, according to the NCCN conversion table. As a result, the patient on a 12 mg/day hydromorphone tablet developed daytime sleepiness, while the patient on a 14 mg/day hydromorphone tablet did not experience daytime sleepiness.

According to the conversion ratio suggested in this study, the optimal dose of oral methadone for switching from a 12 mg/day hydromorphone tablet would be 14.4 mg/day, and the optimal dose of oral methadone for switching from a 14 mg/day hydromorphone tablet would be 16.8 mg/day. As only 5 and 10 mg of methadone tablets are approved in Japan, the methadone dose in both cases was 15 mg/day; switching from hydromorphone to methadone, according to the conversion ratio suggested in this study, was impossible. If lower doses of methadone formulations are introduced, it will allow for more detailed methadone dose adjustments, thus making methadone administration safer.

Regarding the methadone administration frequency, the NCCN conversion table classifies doses of 15, 30, or 45 mg/day, assuming that methadone is administered three times a day. However, in this study, patients who were administered methadone twice a day or low doses of methadone once a day also had 24-hour analgesia. This may be due to the long half-life of methadone. Therefore, it is expected that a low-dose methadone formulation will allow methadone to be administered based on usage and dose according to the conversion ratio suggested in this study, thus preventing daytime sleepiness after methadone initiation.

The incidence of adverse events in patients with daytime sleepiness was 25.0%, of which 40.0% resulted in the discontinuation or reduction in methadone dose due to symptoms other than daytime sleepiness. In contrast, none of patients without daytime sleepiness discontinued or reduced their methadone dose owing to adverse events. Thus, daytime sleepiness was the most frequent adverse event associated with methadone and may be useful in predicting other adverse events.

The reduction in pain intensity on day one was significantly higher in patients with daytime sleepiness than in those without daytime sleepiness, although no statistically significant difference was observed in the reduction in pain intensity on days 2, 3, 5, and 7 between the two groups. While inadequately treated pain may contribute to insomnia and poor sleep quality, adequately or excessively treated pain might increase daytime sleepiness.^7^ Therefore, if a significant response to methadone for cancer pain is observed immediately after methadone initiation, it may be preferable to consider a methadone dose reduction based on safety considerations. It should be noted that the decrease in pain intensity was not due to opioid rescue medications use, but due to the analgesic effect of methadone because both patients with and without daytime sleepiness showed a significant reduction in the average usage counts of opioid rescue medications after methadone initiation.

This was a retrospective study, and we determined the occurrence of daytime sleepiness according to patient complaints, as well as medical records by attending physicians, nurses, and pharmacists; thus, we could not assess the intensity of daytime sleepiness or the quality of sleep. In addition, we could not investigate changes in their ability to perform activities of daily living. Therefore, future prospective studies using sleep questionnaires, such as the Epworth Sleepiness Scale or polygraphic recordings, are desirable to validate our findings.

## Conclusions

This study suggests that when methadone dose is high in comparison to pretreatment MEDD, daytime sleepiness develops. Furthermore, to our knowledge, this is the first study to provide the conversion ratio from pretreatment MEDD to oral methadone, which shows a sufficient analgesic effect without causing daytime sleepiness. Although methadone is effective for intractable cancer pain, data on its safe use remain unavailable. Therefore, our findings will be beneficial for safe and effective cancer pain treatment.

## Abbreviations Used

γ-GTP: γ-glutamyl transpeptidase
ALT: alanine transaminase
AST: aspartate transaminase
BMI: body mass index
BUN: blood urea nitrogen
CI: confidence interval
ECOG PS: Eastern Cooperative Oncology Group Performance Status
eGFR: estimated glomerular filtration rate
IQR: interquartile range
MEDD: morphine-equivalent daily dose
NCCN: National Comprehensive Cancer Network
NMDA: N-methyl-D-aspartate
NRS: numerical rating scale
NSAIDs: nonsteroidal anti-inflammatory drugs
OR: odds ratio
ROC: receiver operator characteristic
Scr: serum creatinine
SD: standard deviation
SNRI: serotonin-norepinephrine reuptake inhibitor
VRS: verbal rating scale

## References

[1] Matthes HW, Maldonado R, Simonin F, et al. Loss of morphine-induced analgesia, reward effect and withdrawal symptoms in mice lacking the mu-opioid-receptor gene. Nature 1996;383(6603):819–823; doi: 10.1038/383819a0 8893006

[2] Cronin A, Keifer JC, Baghdoyan HA, et al. Opioid inhibition of rapid eye movement sleep by a specific mu receptor agonist. Br J Anaesth 1995;74(2):188–192; doi: 10.1093/bja/74.2.188 7696070

[3] Zhou J, Ma R, Jin Y, et al. Molecular mechanisms of opioid tolerance: From opioid receptors to inflammatory mediators (Review). Exp Ther Med 2021;22(3):1004; doi: 10.3892/etm.2021.10437 34345286 PMC8311239

[4] Haumann J, Geurts JW, van Kuijk SM, et al. Methadone is superior to fentanyl in treating neuropathic pain in patients with head-and-neck cancer. Eur J Cancer 2016;65:121–129; doi: 10.1016/j.ejca.2016.06.025 27494037

[5] Davis AM, Inturrisi CE. d-Methadone blocks morphine tolerance and N-methyl-D-aspartate-induced hyperalgesia. J Pharmacol Exp Ther 1999;289(2):1048–1053. 10215686

[6] Nicholson AB, Watson GR, Derry S, et al. Methadone for cancer pain. Cochrane Database Syst Rev 2017;2(2):CD003971; doi: 10.1002/14651858.CD003971.pub4 28177515 PMC6464101

[7] Baldassarri SR, Beitel M, Zinchuk A, et al. Correlates of sleep quality and excessive daytime sleepiness in people with opioid use disorder receiving methadone treatment. Sleep Breath 2020;24(4):1729–1737; doi: 10.1007/s11325-020-02123-z 32556918 PMC7680294

[8] Sharkey KM, Kurth ME, Anderson BJ, et al. Assessing sleep in opioid dependence: A comparison of subjective ratings, sleep diaries, and home polysomnography in methadone maintenance patients. Drug Alcohol Depend 2011;113(2–3):245–248; doi: 10.1016/j.drugalcdep.2010.08.007 20850231 PMC3025068

[9] Wang D, Teichtahl H, Goodman C, et al. Subjective daytime sleepiness and daytime function in patients on stable methadone maintenance treatment: Possible mechanisms. J Clin Sleep Med 2008;4(6):557–562. 19110885 PMC2603533

[10] Walker JM, Farney RJ, Rhondeau SM, et al. Chronic opioid use is a risk factor for the development of central sleep apnea and ataxic breathing. J Clin Sleep Med 2007;3(5):455–461. 17803007 PMC1978331

[11] Berry RB. Central apnea during stage 3,4 sleep. J Clin Sleep Med 2007;3(1):81–82. 17557459

[12] Eckert DJ, Jordan AS, Merchia P, et al. Central sleep apnea: Pathophysiology and treatment. Chest 2007;131(2):595–607; doi: 10.1378/chest.06.2287 17296668 PMC2287191

[13] Bruera E, Palmer JL, Bosnjak S, et al. Methadone versus morphine as a first-line strong opioid for cancer pain: A randomized, double-blind study. J Clin Oncol 2004;22(1):185–192; doi: 10.1200/JCO.2004.03.172 14701781

[14] Paul SM, Zelman DC, Smith M, et al. Categorizing the severity of cancer pain: Further exploration of the establishment of cutpoints. Pain 2005;113(1–2):37–44; doi: 10.1016/j.pain.2004.09.014 15621362

[15] Reddy A, Vidal M, Stephen S, et al. The conversion ratio from intravenous hydromorphone to oral opioids in cancer patients. J Pain Symptom Manage 2017;54(3):280–288; doi: 10.1016/j.jpainsymman.2017.07.001 28711751 PMC13267606

